# ASA Suppresses PGE_2_ in Plasma and Melanocytic Nevi of Human Subjects at Increased Risk for Melanoma

**DOI:** 10.3390/ph13010007

**Published:** 2020-01-02

**Authors:** Amir Varedi, Hafeez Rahman, Dileep Kumar, Jonathan L. Catrow, James E. Cox, Tong Liu, Scott R. Florell, Kenneth M. Boucher, Nwanneka Okwundu, William J. Burnett, Matthew W. VanBrocklin, Douglas Grossman

**Affiliations:** 1 Huntsman Cancer Institute, University of Utah Health Sciences Center, Salt Lake City, UT 84112, USA; Amir.Varedi@gmail.com (A.V.); Hafeez.Rahman@hci.utah.edu (H.R.); Dileep.Kumar@hci.utah.edu (D.K.); Tong.Liu@hci.utah.edu (T.L.); Kenneth.Boucher@hci.utah.edu (K.M.B.); Nwanneka.Okwundu@hci.utah.edu (N.O.); William.Burnett@hci.utah.edu (W.J.B.); Matthew.Vanbrocklin@hci.utah.edu (M.W.V.); 2 Health Science Center Cores, University of Utah Health Sciences Center, Salt Lake City, UT 84112, USA; Leon.Catrow@utah.edu (J.L.C.); jcox@cores.utah.edu (J.E.C.); 3 Department of Biochemistry, University of Utah, Salt Lake City, UT 84112, USA; 4 Department of Dermatology, University of Utah Health Sciences Center, Salt Lake City, UT 84132, USA; Scott.Florell@hsc.utah.edu; 5 Department of Medicine, University of Utah Health Sciences Center, Salt Lake City, UT 84132, USA; 6 Department of Oncological Sciences, University of Utah, Salt Lake City, UT 84112, USA; 7 Department of Surgery, University of Utah Health Sciences Center, Salt Lake City, UT 84132, USA

**Keywords:** aspirin, salicylate, PGE_2_, AMPK, plasma, nevi

## Abstract

Potential anti-inflammatory and anticarcinogenic effects of aspirin (ASA) may be suitable for melanoma chemoprevention, but defining biomarkers in relevant target tissues is prerequisite to performing randomized controlled chemoprevention trials. We conducted open-label studies with ASA in 53 human subjects with melanocytic nevi at increased risk for melanoma. In a pilot study, 12 subjects received a single dose (325 mg) of ASA; metabolites salicylate, salicylurate, and gentisic acid were detected in plasma after 4–8 h, and prostaglandin E2 (PGE_2_) was suppressed in both plasma and nevi for up to 24 h. Subsequently, 41 subjects received either 325 or 81 mg ASA (nonrandomized) daily for one week. ASA metabolites were consistently detected in plasma and nevi, and PGE_2_ levels were significantly reduced in both plasma and nevi. Subchronic ASA dosing did not affect 5” adenosine monophosphate-activated protein kinase (AMPK) activation in nevi or leukocyte subsets in peripheral blood, although metabolomic and cytokine profiling of plasma revealed significant decreases in various (non-ASA-derived) metabolites and inflammatory cytokines. In summary, short courses of daily ASA reduce plasma and nevus PGE_2_ and some metabolites and cytokines in plasma of human subjects at increased risk for melanoma. PGE_2_ may be a useful biomarker in blood and nevi for prospective melanoma chemoprevention studies with ASA.

## 1. Introduction

Aspirin (acetylsalicylic acid, ASA) is administered at dosages of 300–1200 mg for acute analgesia or chronic inflammatory disorders and daily dosages as low as 81 mg for prevention of cardio-/cerebrovascular disease [1]. Its low cost, safety profile, and anti-inflammatory activities make it attractive as a cancer prevention agent. ASA use has been associated with reduced incidence of ovarian cancer [2], hepatocellular cancer [3], prostate cancer [4], and breast cancer [5] in nonrandomized retrospective studies and has demonstrated risk reduction for colon cancer in multiple prospective randomized controlled trials [6]. Daily ASA use, however, is not recommended for adults without increased risk of cancer or cardio-/cerebrovascular events due to potential side effects such as gastric ulcer and bleeding. For example, in the ASPREE trial in healthy elderly subjects, there was no reduction in cancer-related mortality [7] but a near 40% increased risk of major bleeding [8] in those randomized to 100 mg daily ASA vs. placebo. In addition to its anti-inflammatory actions, multiple mechanisms have been proposed underlying the chemopreventive effects of ASA in high-risk subjects, which include suppression of prostaglandin E2 (PGE_2_) synthesis [9], inhibition of nuclear factor-κB [10], and activation of the 5′ adenosine monophosphate-activated protein kinase (AMPK) [11].

There are conflicting reports regarding the efficacy of ASA for melanoma prevention [12], and these prior studies have been primarily retrospective and nonrandomized in nature. There has never been a prospective randomized controlled trial of daily ASA in subjects at increased risk for melanoma. Establishing a dosage regimen that will impact biomarkers related to disease risk in relevant target tissues is prerequisite to performing a randomized controlled prevention trial in high-risk subjects. This is particularly important since some ASA chemoprevention studies did not show a dose-dependent relationship with cancer reduction [2,6], and the minimal dose required to maintain modulation of downstream markers may differ for particular biomarkers and target tissues. The most studied ASA biomarker is cyclooxygenase-2 (COX-2)-mediated generation of PGE_2_, which may be relevant to melanoma development given its induction in the skin [13,14] and melanocytes [15] upon UV exposure, its suppression of skin immune responses [16], the upregulation of COX-2 during melanoma progression [17,18,19], and the role of PGE_2_ in promoting angiogenesis [20,21], cell migration [22], and invasion [23]. The metabolism of ASA in the bloodstream and urine following oral ingestion is well-characterized [24], but studies detecting ASA-derived metabolites have generally been limited to healthy adults [25,26,27,28,29,30], and ASA metabolites other than salicylate were not reported. Reduction of PGE_2_ levels following ASA administration has been shown in urine [9] and tissues of the gastrointestinal tract [31,32,33]. ASA metabolism has not been reported in subjects at risk for melanoma or in potential melanoma precursor tissues such as melanocytic nevi (moles).

Here, we examined ASA-mediated suppression of PGE_2_ and ASA-derived metabolites in plasma and nevi, obtained prior to and following several ASA dosing regimens, from subjects at increased risk for melanoma. We then conducted metabolomic and inflammatory cytokine profiling for potential additional biomarkers in plasma.

## 2. Results

### 2.1. Summary of Human Subject Enrollment

**Figure 1:**
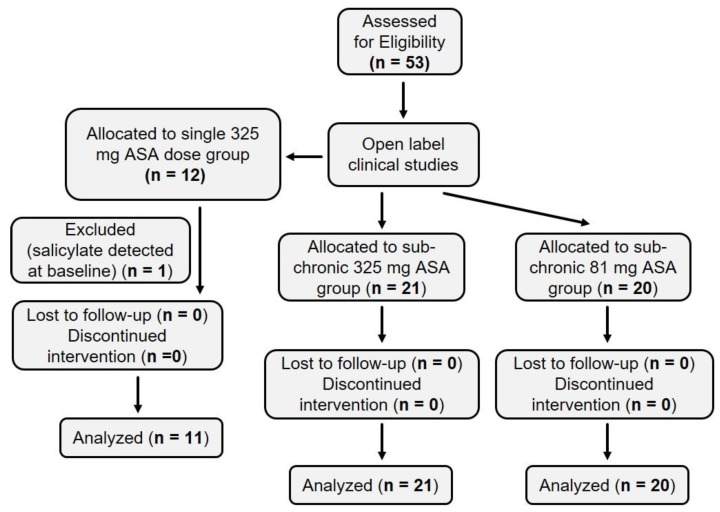
CONSORT flowchart. A total of 53 subjects were eligible and enrolled. Twelve subjects were enrolled in the single 325 mg acetylsalicylic acid (ASA) cohort, and one subject was excluded because salicylate was detected in the baseline (pre-ASA) plasma sample. Twenty-one and 20 subjects were enrolled in the sub-chronic 325 mg and 81 mg ASA cohorts, respectively. All data from these 41 subjects were analyzed.

Figure 1 depicts a flowchart indicating the enrollemnt of human suibjects described in this study. In all cases, studies were open-label and performed in nonrandomized fashion.

### 2.2. Three ASA Metabolites Detected in Plasma Following Single Dose of ASA

**Figure 2:**
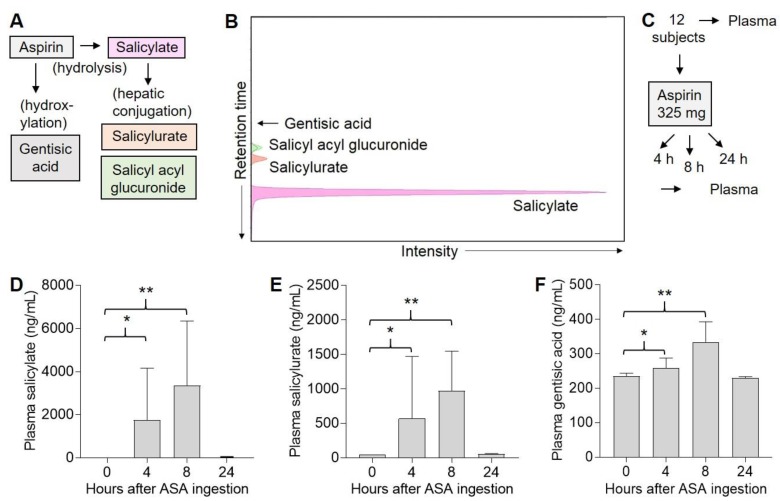
Detection of ASA metabolites in human plasma. (**A**), Metabolism of ASA. (**B**), Liquid chromatography–mass spectrometry (LC–MS) detection of ASA metabolite standards. (**C**), Experimental design. Human subjects were each given a single 325 mg dose of ASA, and blood was drawn both before (0 h) and either 4 (n = 5), 8 (n = 3), or 24 (n = 3) h later. Plasma was isolated from blood, and 0.5 mL was extracted for LC–MS analysis. (**D**), Plasma salicylate levels. Error bars indicate SEM. * *p* = 0.02, ** *p* = 0.001. (**E**), Plasma salicylurate levels. Error bars indicate SEM. * *p* = 0.07, ** *p* < 0.001. (**F**), Plasma gentisic acid levels. Error bars indicate SEM. * *p* = 0.03, ** *p* < 0.001. Note: ASA and salicyl acyl glucuronide were not detected in any of the samples.

ASA is rapidly hydrolyzed to salicylate, or may be hydroxylated to gentisic acid; in the liver, salicylate is conjugated to salicylurate and salicyl acyl glucuronide (Figure 2A). We optimized the separation of various ASA metabolites by liquid chromatography–mass spectrometry (LC–MS) (Figure 2B). In a pilot study, 12 subjects were given a single 325 mg oral dose of ASA, and plasma samples were obtained both before (0 h) and either 4, 8, or 24 h after ASA exposure (Figure 2C). Consistent with its short half-life [34], we did not detect ASA in any of the plasma samples. We detected salicylate at 0 h in one subject’s plasma, who presumably had been taking ASA (or ASA-containing product), and this subject was excluded from further analysis. No subjects were lost to follow up. Among the remaining 11 subjects, significant levels of plasma salicylate were detected over background in two of five subjects after 4 h, in three of three subjects after 8 h, and in none of three subjects at 24 h (Figure 2D). Similarly, significant levels of plasma salicylurate were detected over background in two of five subjects after 4 h, in three of three subjects after 8 h, and in none of three subjects at 24 h (Figure 2E). Significant levels of plasma gentisic acid were detected over background in three of five subjects after 4 h, in three of three subjects after 8 h, and in none of three subjects at 24 h (Figure 2F). Finally, we did not detect significant levels of plasma salicyl acyl glucuronide in any of these samples at any of the time points (not shown). Thus, various ASA-derived metabolites could be detected in plasma over 4–8 h in some subjects following ingestion of a single 325 mg dose of ASA, but none were detectable by 24 h.

### 2.3. Suppression of PGE_2_ in Plasma and Nevi Following Single Dose of ASA

**Figure 3:**
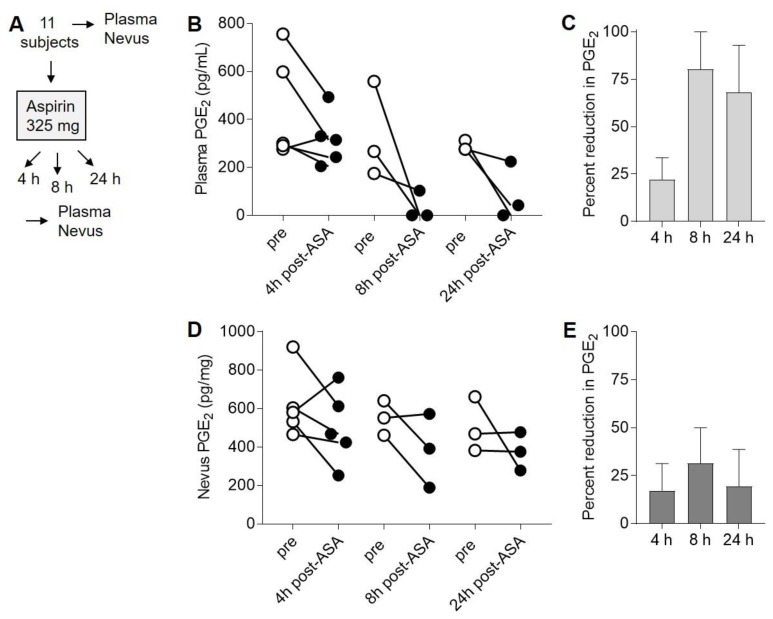
Suppression of prostaglandin E2 (PGE_2_) in plasma and nevi following single dose of ASA. (**A**), Experimental design. Human subjects were each given a single 325 mg dose of ASA, and blood and nevus samples were obtained both before (pre) and either 4 (n = 5), 8 (n = 3), or 24 (n = 3) h later. (**B**), Plasma PGE_2_ levels before (open circles) and after (filled circles) aspirin treatment. (**C**), Average percent reduction in plasma PGE_2_ at each time point from (**B**). Error bars represent SEM. *p* = 0.09, ANOVA. (**D**), PGE_2_ levels in nevi taken before (open circles) and after (filled circles) ASA treatment. (**E**), Average percent reduction in nevus PGE_2_ at each time point from (**D**). Error bars represent SEM. *p* = 0.8, ANOVA. For the ANOVA analyses, the log ratio was first calculated for each subject, and then the log ratios were compared between groups.

Next, we examined the levels of PGE_2_ in both plasma and nevi obtained from these 11 subjects both before (0 h) and either 4, 8, or 24 h after ASA exposure (Figure 3A). Determination of PGE_2_ from these samples by LC–MS revealed reduction in plasma PGE_2_ levels in four of five subjects at 4 h, in three of three subjects after 8 h, and in three of three subjects at 24 h (Figure 3B). The average percent reduction in plasma PGE_2_ at each time point ranged from 25% to 75% (Figure 3C), and there was not a significant (*p* = 0.09, ANOVA) difference in PGE_2_ reduction between the three time points. Because we initially encountered technical difficulties analyzing ASA metabolites and PGE_2_ in homogenized nevi by LC–MS, we used ELISA for all subsequent measurements of PGE_2_. Nevus PGE_2_ levels were reduced in four of five subjects at 4 h, in two of three subjects after 8 h, and in one of three subjects at 24 h (Figure 3D). The average percent reduction in nevus PGE_2_ at each time point ranged from 20% to 30% (Figure 3E), and there was not a significant (*p* = 0.8, ANOVA) difference in PGE_2_ reduction between the three time points. Thus, there was variability between subjects and time points in the magnitude of ASA-mediated PGE_2_ suppression in both plasma and nevi, and there did not appear to be an optimal time point for observing PGE_2_ reduction following a single dose of ASA in the pilot study.

### 2.4. ASA Suppresses PGE_2_ in Nevi Expressing Mutant BRAF^V600E^

Prior studies have implicated RAF–MAPK signaling in COX-2 upregulation and PGE_2_ synthesis [35,36]. Given that the majority (>80%) of melanocytic nevi express mutant BRAF (usually BRAF^V600E^) [37], and a prior chemoprevention study of ASA and colon cancer concluded that ASA may only be protective against BRAF wild-type colon cancers [38], we examined whether ASA-mediated suppression of PGE_2_ in nevi was related to BRAF mutation status. Sections from seven nevi that demonstrated reduction in PGE_2_ following ASA in the pilot study were stained with a BRAF^V600E^-specific antibody. Six of seven (86%) specimens were positive for BRAF^V600E^ by immunostaining (Figure S1), indicating that BRAF-mutant nevi are susceptible to ASA-mediated suppression of PGE_2_.

### 2.5. Four ASA Metabolites Detected in Plasma Following Sub-Chronic ASA Dosing

**Figure 4:**
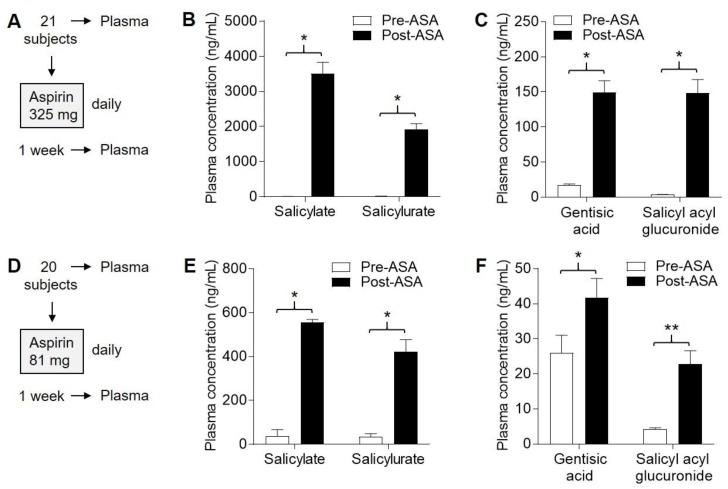
Detection of ASA metabolites in plasma following 1 week of daily ASA exposure. Human subjects were each given ASA daily for one week, and tissue samples were obtained both before (pre) and after (post) this period. (**A**), Protocol for 325 mg dosing. (**B**), Levels of salicylate and salicylurate in plasma in the 325 mg cohort. * *p* < 0.0001, paired *t*-test. (**C**), Levels of gentisic acid and salicyl acyl glucuronide in plasma in the 325 mg cohort. * *p* < 0.0001, paired *t*-test. (**D**), Protocol for 81 mg dosing. (**E**), Levels of salicylate and salicylurate in plasma in the 81 mg cohort. * *p* < 0.0001, paired *t*-test. (**F**), Levels of gentisic acid and salicyl acyl glucuronide in plasma in the 81 mg cohort. * *p* = 0.04, ** *p* < 0.0001, paired *t*-tests.

Next, we conducted an open-label (subchronic dosing) study involving an additional 41 participants who were given either 325 mg (Figure 4A) or 81 mg (Figure 4D) ASA daily for one week. The two dosage arms were similar for sex, age, and weight distributions among the subjects (Table S1). Similar to the pilot study, each subject served as their own control, and samples were obtained before and after ASA exposure. Blood samples were obtained prior to ASA exposure and approximately 8–12 h following the seventh ASA dose. No subjects were lost to follow up. In the 325 mg cohort, plasma salicylate (1061–6299 ng/mL) and salicylurate (673–3355 ng/mL) were detected in 21/21 (100%) subjects (Figure 4B), gentisic acid (39–283 ng/mL) was detected in 20/21 (95%) subjects, and salicyl acyl glucuronide (31–396 ng/mL) was detected in 21/21 (100%) subjects (Figure 4C) following ASA exposure and were highly significant (*p* < 0.0001 for each metabolite, paired *t*-tests). Plasma levels of salicylate, salicylurate, and gentisic acid were similar to those detected in the pilot study based on a single 325 mg dose (Figure 2D–F). It is likely that successful detection of salicyl acyl glucuronide, as well as the other three ASA metabolites in > 95% of subjects, was due to accumulation of salicylate (which has the longest half-life) in the blood after repeated daily ASA exposure.

In the 81 mg cohort, plasma salicylate (36–1408 ng/mL) was detected in 19/20 (95%) subjects, salicylurate (101–824 ng/mL) was detected in 19/20 (95%) subjects (Figure 4E), gentisic acid (15–96 ng/mL) was detected in 15/20 (75%) subjects, and salicyl acyl glucuronide (7.3–60 ng/mL) was detected in 19/20 (95%) subjects (Figure 4F) following ASA exposure and were significant (*p* = 0.04 for gentisic acid, and *p* < 0.0001 for other metabolites). In summary, all four ASA metabolites were detected in plasma in both cohorts, but plasma levels of all four metabolites on average were approximately four- to fivefold lower in the 81 mg compared to the 325 mg cohort.

### 2.6. Detection of ASA Metabolites in Nevi Following Subchronic ASA Dosing

**Figure 5:**
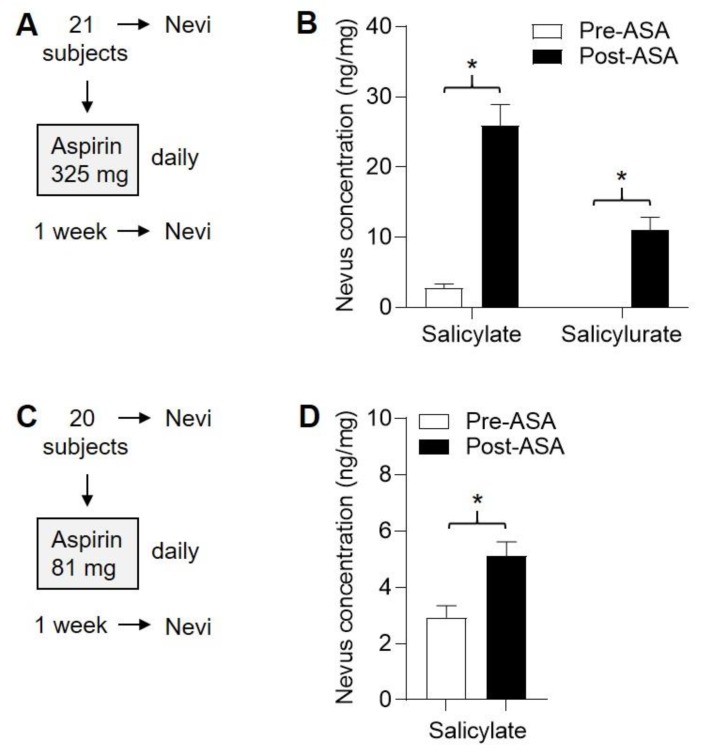
Detection of ASA metabolites in nevi following 1 week of daily ASA exposure. Human subjects were each given ASA daily for one week, and nevi were obtained both before (pre) and after (post) this period. (**A**), Protocol for 325 mg dosing. (**B**), Levels of salicylate and salicylurate in nevi in the 325 mg cohort. * *p* < 0.0001, paired *t*-test. (**C**), Protocol for 81 mg dosing. (**D**), Levels of salicylate and salicylurate in nevi in the 81 mg cohort. * *p* < 0.01, paired *t*-test. Salicylurate was detected in nevi of only two subjects from the 81 mg cohort (not shown). Gentisic acid and salicyl acyl glucuronide were not detected in any of the nevi.

Nevi were obtained prior to ASA exposure and approximately 8–12 h following the seventh ASA dose. From the 325 mg cohort, nevi were also obtained (Figure 5A), and nevus salicylate (6.6–58 ng/mg) and salicylurate (1.3–23 ng/mg) were detected in 21/21 (100%) and 19/21 (90%) subjects (Figure 5B), respectively, following ASA exposure and were highly significant (*p* < 0.0001 for each metabolite, paired *t*-tests). From the 81 mg cohort (Figure 5C), nevus salicylate (2.4–10.2 ng/mg) was detected in 20/20 (100%) subjects following ASA exposure and was significant (Figure 5D, *p* < 0.01, paired *t*-tests). Salicylurate (1.7–2.3 ng/mg) was detected in 2/20 (10%) subjects (not shown). Gentisic acid and salicyl acyl glucuronide were not detected in any nevi from either cohort.

### 2.7. Suppression of PGE_2_ in Plasma and Nevi Following Subchronic ASA Dosing

**Figure 6:**
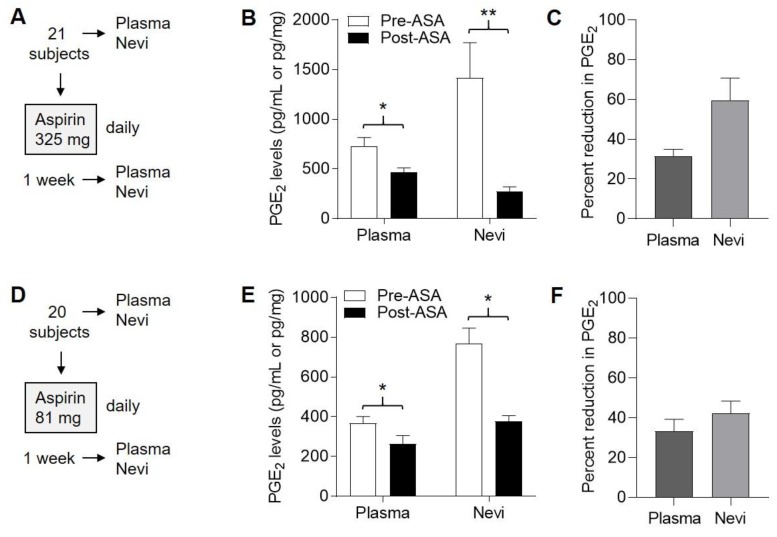
Suppression of PGE_2_ in plasma and nevi following 1 week of daily ASA exposure. Human subjects were each given ASA daily for one week, and blood and nevus samples were obtained both before (pre) and after (post) this period. (**A**), Protocol for 325 mg dosing. (**B**), Levels of PGE_2_ in plasma and nevi in the 325 mg cohort. Error bars represent SEM. * *p* < 0.0001, ** *p* = 0.004, paired *t*-tests. (**C**), Average percent reduction in PGE_2_ levels from (**B**). Error bars represent SEM. (**D**), Protocol for 81 mg dosing. (**E**), Levels of PGE_2_ in plasma and nevi in the 81 mg cohort. * *p* < 0.0001, paired *t*-tests. (**F**), Average percent reduction in PGE_2_ levels from (**E**). Error bars represent SEM.

Plasma PGE_2_ levels ranged from 332 to 1642 (mean 731) pg/mL prior to ASA exposure in the 325 mg cohort (Figure 6A) and were significantly reduced (*p* < 0.0001, paired *t*-test) following one week of ASA exposure (Figure 6B), on the order of 30–35% (Figure 6C). In the 81 mg cohort (Figure 6D), plasma PGE_2_ levels ranged from 126 to 721 (mean 699) pg/mL prior to ASA exposure and were significantly reduced (*p* < 0.0001, paired *t*-test) following one week of ASA exposure (Figure 6E), on the order of 25–40% (Figure 6F). Thus, similar percent reduction in plasma PGE_2_ levels was achieved in both the 325 mg and 81 mg cohorts.

From the 325 mg cohort (Figure 6A), nevus PGE_2_ levels ranged from 153 to 5615 (mean 1417) pg/mg prior to ASA exposure and were significantly reduced (*p* < 0.0001, paired *t*-test) following one week of ASA exposure (Figure 6B), on the order of 50–70% (Figure 6C). In the 81 mg cohort (Figure 6D), nevus PGE_2_ levels ranged from 234 to 1611 (mean 769) pg/mg prior to ASA exposure and were significantly reduced (*p* < 0.0001, paired *t*-test) following one week of ASA exposure (Figure 6E), on the order of 35–50% (Figure 6F). Thus, although we observed considerable variation in nevus levels of PGE_2_ among all the subjects, paired analyses revealed that PGE_2_ levels were significantly reduced in nevi in both cohorts, with a greater percent reduction observed in the 325 mg vs. 81 mg cohort.

### 2.8. Variable Effect on AMPK Activation in Nevi Following Subchronic ASA Dosing

ASA activates AMPK [11], which modulates the mTOR pathway in various cancer cell types [39,40] and may in part mediate its anticancer effects. We previously reported that ASA-mediated inhibition of cellular migration and pigmentation in both melanoma cells and melanocytes was dependent on activation of AMPK [22]. We examined nevi in both cohorts for expression of activated (phosphorylated) AMPK. In comparing normalized phosphorylated AMPK levels in paired (pre- and post-ASA) nevus samples, ASA-mediated AMPK activation was evident (increased) in about a quarter and half of subjects’ nevi in the 325 mg and 81 mg cohorts, respectively, and was unchanged or decreased in the remainder (Figure S2). Thus, there was considerable intersubject variability in response of AMPK in nevi to sub-chronic ASA treatment.

### 2.9. ASA Reduces Inflammatory Cytokines without Affecting Leukocyte Subsets

We profiled whole blood leukocytes and plasma cytokines in both subchronic ASA cohorts. For both cohorts, no significant aggregate changes were observed in whole blood counts of total leukocytes, lymphocytes, monocytes, or granulocytes in comparing pre- and post-ASA samples (Figure S3). Levels of most cytokines were unchanged in comparing pre- and post-ASA samples from the 325 mg sub-chronic cohort, although IL-7, BDNF, and PDGF-BB were significantly reduced (*p* < 0.05, paired *t*-tests) following ASA exposure (Figure S4). In the 81 mg subchronic cohort, we observed significant (*p* < 0.05, paired *t*-tests) reductions following ASA exposure in pleiotropic factors IL-9, IL-13, and IL-15, along with proinflammatory cytokines IFN-α, IFN-γ, IL-6, and TNF-α (Figure S5).

### 2.10. Metabolites Suppressed by ASA

Finally, we profiled up metabolites in plasma from both subchronic ASA cohorts. Approximately 60–80 different metabolites were detectable in each sample, with 2-hydroxybenzoate (an ASA-derived metabolite) serving as an internal control. Levels of most metabolites were unchanged in comparing pre- and post-ASA samples from the 325 mg subchronic cohort, although 2-hydroxybenzoate was significantly increased (*p* < 0.001, paired t test) while D-glucose, L-aspartate, and cholesterol (*p* < 0.01, paired *t*-tests), and isocitrate, L-glutamate, sorbitol, ribitol, myo-inositol, oleic acid, 2-hydroxybutyrate, hypotaurine, 2’,3’-biphosphoglycerate, benzoate, and campesterol, were significantly reduced (*p* < 0.05, paired *t*-tests) following ASA exposure (Figure S6, top). In the 81 mg subchronic cohort, levels of almost all detectable metabolites were unchanged, although 2-hydroxybenzoate was significantly increased (*p* < 0.01, paired *t*-test), while L-valine was significantly reduced (*p* < 0.05, paired *t*-test) following ASA exposure (Figure S6, bottom). Levels of 2-hydroxyglutarate were increased in the 325 mg cohort (Figure S6, top) and decreased in the 81 mg cohort (Figure S6, bottom) following ASA exposure, but neither of these changes reached statistical significance. Finally, pathway analysis did not reveal significant alteration with ASA exposure in tested metabolic pathways (not shown).

## 3. Discussion

We completed open-label studies using two conventional ASA doses in subjects at increased risk for melanoma. Rather than a placebo-controlled experimental design, we analyzed both pre- and post-ASA-exposure tissue specimens, with each subject serving as their own internal control so that paired analyses could be performed. We observed a robust suppression of PGE_2_ in both plasma and nevi following both single-dose and subchronic ASA regimens. Although inflammatory profiling did not demonstrate any significant changes in peripheral blood leukocyte subsets, multiple cytokines were reduced, particularly in the 81 mg cohort. Metabolomic profiling demonstrated reduction in multiple metabolites, particularly in the 325 mg cohort. Additional studies will be required to validate these as potential ASA-modulated biomarkers in addition to PGE_2_. We did not demonstrate significant reductions in plasma 2-hydroxyglutarate following ASA exposure, in contrast to the findings of Leisenfeld et al. [25]; however, in that study, healthy adults were given 325 mg ASA daily for 2 months compared to the 1-week dosing period used here.

### 3.1. Quantitative Detection of ASA Metabolites

There are few studies reporting plasma levels of ASA metabolites at specific time points in human subjects. Most prior work investigating ASA metabolism was focused on validation of detection techniques without human subject involvement [41,42,43] or reported fold changes or intensity values which cannot easily be translated to actual concentrations [25,26,27,28]. The plasma levels of salicylate we detected using LC–MS methodology are in line with those of other studies [29,30] in which this metabolite was measured following a single ASA dose. Neither of these studies reported on ASA metabolites other than salicylate, as we have done here, or the metabolite levels in plasma following subchronic dosing. Two studies [29,44] reported detection of ASA and salicylate (1000–2000 ng/cm^2^) in human tape-stripped skin 1 h following a single 500 mg oral dose of ASA. It is unclear if ASA and its metabolites diffuse from plasma to the avascular stratum corneum or if the process of repeated tape-stripping disrupts the epidermal barrier allowing contamination of stratum corneum with plasma. We previously reported detection of salicylate (200–300 ng/g) in whole mouse skin 4 h following oral gavage with 0.4 mg ASA [22]. To our knowledge, there are no studies detecting ASA metabolites in whole human skin, which may be problematic due to the lack of a suitable solubilization procedure compatible with extraction for LC–MS analysis. We successfully detected salicylate in all nevus samples following ASA exposure, with levels being 3- to 5-fold higher in samples from subjects in the 325 (compared to 81 mg) cohort.

### 3.2. Anti-Inflammatory Effects of ASA

The anti-inflammatory mechanisms of ASA have not been well defined. Claria and Serhan [45] showed that ASA induces synthesis of various lipid eicosanoids in neutrophils that may impede leukocyte trafficking. Morris et al. [46] demonstrated that a 10-day course of 75 mg daily ASA reduced polymorphonuclear leukocyte and macrophage accumulation in cantharidin-induced skin blisters in healthy male subjects. Although we did not observe any effect of ASA on total white blood cell counts or leukocyte subsets, we did observe significant reduction in plasma levels of several proinflammatory interleukins and interferons that have been implicated in malignant progression [47]. Although many of the observed statistically significant reductions in cytokines were small in magnitude (e.g., <25%), it is important to consider that a small change in cytokine concentration in peripheral blood may represent a large change in systemic cytokine levels that may have significant biological implications.

### 3.3. Modulation of PGE_2_ in Relevant Target Tissues

We observed consistent and significant reductions in PGE_2_ levels in both plasma (25–40% reduction) and nevi (35–50% reduction) in our cohorts following subchronic ASA dosing. Boutaud et al. [9] reported a comparable reduction in PGE_2_ in urine in subjects taking 81 mg ASA daily for two weeks. Several studies have shown that oral ASA can suppress PGE_2_ levels in relevant tissues for colon cancer chemoprevention. Sample et al. [31] reported 70% suppression of PGE_2_ levels in rectal mucosa in subjects following 4 weeks of daily (81–650 mg) ASA. Studies by Ruffin et al. [33] and Ferreira et al. [32] demonstrated 24% and 50% reduction of PGE_2_ in colorectal and gastric tissues, respectively, in subjects following short courses of 81 mg ASA; tissue biopsies were obtained 24 h or greater after the last ASA dose, suggesting that ASA-mediated suppression of PGE_2_ is long lived. Similarly, we found that PGE_2_ levels in both plasma and nevi were suppressed for up to 24 h in subjects following a single 325 mg dose.

### 3.4. Looking Toward Future Melanoma Chemoprevention Studies

Nevi are particularly relevant tissues for melanoma chemoprevention since they can be precursors for melanoma [48]. Our findings that PGE_2_ can be suppressed in nevi (including BRAF-mutant nevi) in subjects at increased risk for melanoma suggest that PGE_2_ in nevi may be a useful biomarker and tissue target in future melanoma chemoprevention studies. Indeed, COX-2 expression in nevi and melanoma correlates with melanoma progression [17,18]. While PGE_2_ is an established cancer-relevant biomarker for ASA-mediated chemoprevention [49], the various cytokines and metabolites we identified in this study may also prove to be useful biomarkers, but these need to be validated in a larger, randomized, placebo-controlled trial. Future studies may also examine the effect of ASA on nevi exposed to ultraviolet (UV) radiation, given that human melanocytes produce PGE_2_ upon UV exposure [15].

## 4. Materials and Methods

### 4.1. Human Subjects

This study was approved by the Institutional Review Board (IRB# 94424) of the University of Utah, which determined that this study involved use of an FDA-approved drug for which an Investigational New Drug (IND) application was not required and thus issued an IND exemption, according to 21 CFR 312.2(b). Patients at increased risk for melanoma (with atypical or numerous nevi) were recruited from one of the investigator’s (D.G.) pigmented lesion clinic at the Huntsman Cancer Institute. Subjects not between ages 18–65, or those pregnant or breastfeeding, with recent (<2 weeks) use of NSAIDs or taking anticoagulants, with recent (<1 month) intense sun exposure, or history of peptic ulcer disease, gastrointestinal bleeding, a coagulation disorder, severe asthma, or previous allergic reaction to ASA, were excluded. All patients signed an IRB-approved informed consent form prior to participating. All females of child-bearing potential had a negative urine pregnancy test at the first study visit. Participants who provided two blood samples and two nevi were each compensated $200 after completion of two study visits. Subjects were recruited sequentially, in nonrandomized fashion. There were no serious adverse events. The date range for subject enrollment and follow up was 1/26/17–2/27/19. This study has been registered at ClinicalTrials.gov (NCT04062032).

### 4.2. ASA

ASA was obtained from the University of Utah Hospital pharmacy. For the single-dose study, participants were given a single enteric-coated ASA 325 mg tablet (NDC# 00904201360) and returned at a specified time later for the second visit. For the subchronic dosing study, participants were informed of their dosing assignment, and each was provided seven individually packaged enteric-coated ASA tablets of either 325 mg (NDC# 63739523012) or 81 mg (NDC# 63739522015) to take home. They were instructed to take one tablet orally at approximately the same time every day beginning on the evening of the first study visit and were provided with a log to record the dates and times they took each tablet.

### 4.3. Nevus Tissues

Two nevi (one at each study visit) were obtained from each participant, as described previously [50]. A representative 1 mm slice of each specimen was formalin-fixed and paraffin-embedded, and a hematoxylin/eosin-stained section was later reviewed by a dermatopathologist (S.R.F.). The remaining tissue was placed in a microfuge tube on ice and subsequently stored at −80 °C. Immunostaining for BRAF^V600E^ was performed on deparaffinized nevus sections using clone VE1 antibody (1:600 dilution, Spring Biosciences) as described previously [51].

### 4.4. Blood Collection

Venous blood (5–6 mL) was collected at each study visit in BD Vacutainer™ tubes (K2EDTA 10.8 mg, Thermo Fisher Scientific). A small aliquot was set aside for determination of complete blood counts with differential using a Heska HemaTrue analyzer (Loveland, CO, USA). The remaining whole blood was centrifuged and the plasma was removed by pipetting, aliquotted, and stored at −80 °C.

### 4.5. Detection of ASA Metabolites and PGE_2_ by LC–MS

Detection of ASA metabolites in plasma and nevi by LC–MS is described in the Supplementary Information (Text S1). Quantitation of PGE_2_ in plasma samples by liquid chromatography–mass spectrometry (LC–MS) using a commercial standard was described previously [22].

### 4.6. Detection of PGE_2_ in Plasma and Nevi by ELISA

PGE_2_ content in plasma samples was determined by ELISA using a PGE_2_ assay kit (KGE004B, R&D Systems, Minneapolis, MN, USA) according to the manufacturer’s instructions as described previously [22]. For nevus samples, frozen tissue was cut into sub-millimeter pieces and then homogenized in buffer (RD-556) supplied in the assay kit, using a disposable pestle (Kimble 749521-0590) obtained from Sigma-Aldrich, St. Louis, MO) on ice. After homogenization, nevus tissue lysates were sonicated briefly and then microfuged at 10,000 rpm for 10 min at 4 °C. After centrifugation, supernatants were collected, and protein concentrations were determined using a BCA protein detection kit (Thermo Fisher Scientific, Waltham, MA, USA). PGE_2_ values were normalized to protein content for each sample.

### 4.7. Western Blotting

Nevus fragments were homogenized in RIPA lysis buffer containing 1% NP40 with disposable pestle on ice, then sonicated briefly and microfuged at 10,000 rpm for 15 min at 4 °C. Supernatants were collected, protein concentrations were determined using BCA as above, and then Western blotting was performed with 20 µg of protein lysate as described previously [22].

### 4.8. Cytokine Analysis

A panel of 42 cytokines was simultaneously measured in plasma samples (25 μL) using a ProcartaPlex Multiplex Immunoassay kit (EPXP420-10200-901, Thermo Fisher Scientific) following the manufacturer’s instructions. Briefly, samples were thawed on ice, vortexed, and then microfuged at 10,000 g for 10 min at 4 °C. Samples, standards, and blanks were then added to the multiplexing magnetic capture beads in a 96-well plate and incubated in the dark with shaking at 500 rpm for 2 h at room temperature. Beads were washed twice, and then detection antibody was added for 30 min. Beads were washed again before addition of Streptavidin-PE for 30 min. Two final washes were performed prior to resuspending beads in the supplied reading buffer. Samples were then read on a MAGPIX Luminex scanner (XMAP Technologies, Austin, TX, and data were analyzed using accompanying software (Xponent version 4.2 Build 1705, Austin, TX, USA).

### 4.9. Metabolomics

Untargeted metabolic profiling of plasma samples by gas chromatography–mass spectrometry (GC–MS) was performed as described previously [52], and a brief description is provided in the Supplementary Information (Text S1).

### 4.10. Statistics

Analyses were performed by a statistician (K.M.B.), who was blinded as to subject assignment. For the single-ASA-dose pilot study, the log2 (PGE_2_ post/PGE_2_ pre) was analyzed. Analyses of the single-dose study and associated power calculations were conducted using R software (R Foundation for Statistical Computing, Vienna, Austria). Comparisons between experimental groups of the sub-chronic dosing study were analyzed using students *t*-tests with Prism software (version 7, GraphPad, La Jolla, CA. *p* values of <0.05 were considered significant. The value 1, which was <10% of the smallest non-zero plasma value, was added to both the numerator and denominator of the plasma data prior to analysis because some values were zero. One-way analysis of variance was used to determine if there were differences in mean log ratio by time point. One-sample *t*-tests were used to determine if the mean log ratio differed from zero. The observed standard deviation of log ratios from the single-dose study was used to estimate the sample size needed for 80% power to detect a 50% decrease in PGE_2_ in plasma or 25% decrease in nevi. These correspond to changes of 1 and 0.415 in the log2 ratio. For plasma power calculations, we assume the use of a *t*-test on the log ratio, and SD (log2 ratio) = 3.71 from the single-dose pilot study. A sample size n = 110 is required for 80% power to detect a 50% reduction (at two-sided alpha = 0.05), n = 29 to detect a 75% reduction, and n = 15 to detect an 87% reduction (effect observed in single-dose pilot study). For nevi, a 27% geometric mean reduction was seen in the single-dose pilot study. For nevi power calculations, we assume the use of a *t*-test on the log ratio and SD (log2 ratio) = 0.578 from the single-dose pilot study. A sample size n = 18 is required for 80% power to detect a 25% reduction at two-sided alpha = 0.05. These calculations justify a sample size n = 20 for the subchronic dosing study. For comparisons of demographic factors for the subjects who received 325 vs. 81 mg ASA, Fisher’s Exact test was used for sex, and *t*-tests were used for age and weight.

## Supplementary Materials

The following are available online at https://www.mdpi.com/1424-8247/13/1/7/s1: Text S1: Supplementary methods. Table S1: Demographics of the subchronic cohorts. Figure S1: Immunostaining of nevi for BRAF^V600E^. Figure S2: Lack of effect on nevus AMPK activation in subjects taking ASA. Figure S3: Lack of effect on blood leukocytes in subjects taking ASA. Figure S4: Effect on plasma cytokine levels in subjects taking 325 mg daily ASA. Figure S5: Effect on plasma cytokine levels in subjects taking 81 mg daily ASA. Figure S6: Suppression of metabolites in plasma following 1 week of daily ASA exposure.
