# Supplementary material for: ASA Suppresses PGE2 in Plasma and Melanocytic Nevi of Human Subjects at Increased Risk for Melanoma
Source: Pharmaceuticals (Basel). 2020 Jan 2;13(1):7. doi: 10.3390/ph13010007 (PMC7168893; doi:10.3390/ph13010007)
Supplement: Supplementary file 1 [file pharmaceuticals-13-00007-s001.pdf]

Supplementary information for Varedi et al.

Text S1: Supplementary methods

Detection of ASA metabolites in plasma by LC-MS

For detection of ASA-derived metabolites, calibration curves were made using commercial standards (Sigma) diluted in 50:50 methanol:water + 1% formic acid. Salicylic acid-d4 (10 µg, Cambridge Isotopes) was added to each plasma sample (0.75 mL) which was acidified by addition of 10 uL of 80% phosphoric acid. Oasis HLB 3cc (90 mg, Waters) solid phase extraction cartridges were each wetted with 2 mL methanol and conditioned with 2 mL of 20 mM dipotassium phosphite buffer containing 0.2% phosphoric acid. Samples were added to the cartridges and then washed with 2 mL conditioning solution to remove non-bound interfering metabolites. ASA metabolites were eluted with 1 mL of methanol and dried under vacuum overnight. Each sample was reconstituted with 250 uL of 50:50 methanol:water + 1% formic acid (and a pooled quality control sample) and loaded onto a ZORBAX Eclipse C18 plus 50 x 2.1 mm, 5 um particle column (Agilent Technologies). Fractionation was accomplished using 1% formic acid in water (buffer A) and 1% formic acid in acetonitrile (buffer B) at a flow rate of 0.3 mL/min. Buffer B was initially held at 10% for 0.5 min, then ramped to 60% over 3.5 min, followed by a second ramp to 95% over 1 min. This concentration was held for 1.2 min then returned to the original buffer B concentration over 0.1 min followed by column equilibration for 7 min. Mass spectrometry was performed in the negative mode using a Sciex 6500 Q-Trap with an TurboIon source using optimized source conditions: Curtain Gas 30 L/min, Collision Gas high, IonSpray Voltage -4500 V, Temperature 350° C, Ion Source Gas 1 50 L/min, Ion Source Gas 2 70 L/min. Declustering Potential, Entrance Potential, and Collision Cell Exit Potential for all compounds were set to -50, -10 and -15, respectively. Transitions for salicylic acid-d4 (141.05 → 97.0, collision energy -16 V), salicylic acid (137.02 → 93.0, collision energy -16 V), gentesic acid (153.02 → 108.0, collision energy -10 V), salicyluric acid (194.04 → 150.0, collision energy -10), and salicyl acyl glucuronide (313.050 → 136.9, collision energy -12) were monitored via multiple reaction monitoring. Quantitative data analysis was conducted using Sciex MultiQuant software.

Detection of ASA metabolites in nevi by LC-MS

Each nevus fragment was processed for 30 s in a beadmill tube with ceramic beads and 1 mL water for homogenization. Salicylic acid-d4 was added to each sample for a final concentration of 150 ng/mL. Samples were acidified using 1 uL of concentrated phosphoric acid (80%). Samples were prepared using solid phase extraction (SPE) with Oasis HLB 1cc (30 mg) extraction cartridges, eluted in methanol, and then subjected to fractionation and mass spectrometry as described above.

Metabolomics

Untargeted metabolic profiling of plasma samples by gas chromatography-mass spectrometry (GC-MS) was performed. Briefly, metabolites were extracted using cold 90% methanol solution containing the internal standard d4-succinic acid (Sigma 293075). A second standard was then added (d27-myristic acid, CDN Isotopes: D-1711). Absolute concentrations were not determined for the metabolites, but instead the area under the curve was used to obtain relative measures of concentrations compared to the standards. GC-MS analysis was performed with an

Agilent 7200 GC-QTOF and an Agilent 7693A automatic liquid sampler. Data was collected and analyzed using MassHunter software (Agilent). Metabolite identity was established using a combination of an in-house metabolite library developed using pure purchased standards and other libraries (NIST, Fiehn). Pathway mapping was performed using freely available software (Metaboanalsyt 3.0, http://www.metaboanalyst.ca).

Supplementary table

Supplementary Table S1. Demographics of the sub-chronic cohorts

**Subjects**	**ASA dose**	**Females / Males**	**P value***	**Age, mean (range)**	**P value#**	**Weight, mean (range)**	**P value#**
21	325 mg	12 / 9		43.4<br>(22-64)		182.6<br>(125-300)	
20	81 mg	10 / 10	0.76	43.5<br>(25-60)	0.91	178.5<br>(131-230)	0.90

*Fisher's Exact test.

#2-sided t test.

Supplementary figures

Figure S1

A BRAF^{V600E} by immuno-staining

Nevus samples tested	7
BRAF^{V600E}-positive	6
BRAF^{V600E}-negative	1

B

C

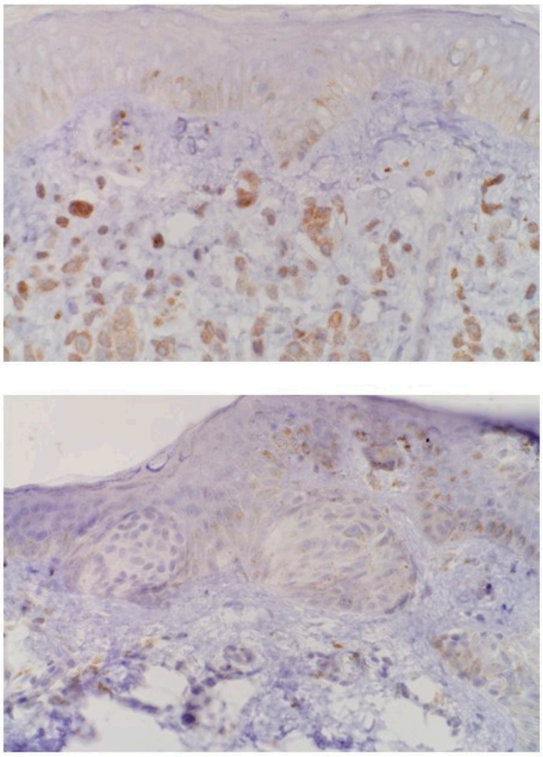

Fig. S1 Immunostaining of nevi for BRAF-^{V600E}. **A**, Summary of staining results for seven nevi in which PGE_2 levels decreased in subjects following a single oral dose of 325 mg ASA. **B**, Representative photograph of nevus staining positive for BRAF^{V600E}. **C**, Nevus negative for expression of BRAF^{V600E}.

Figure S2

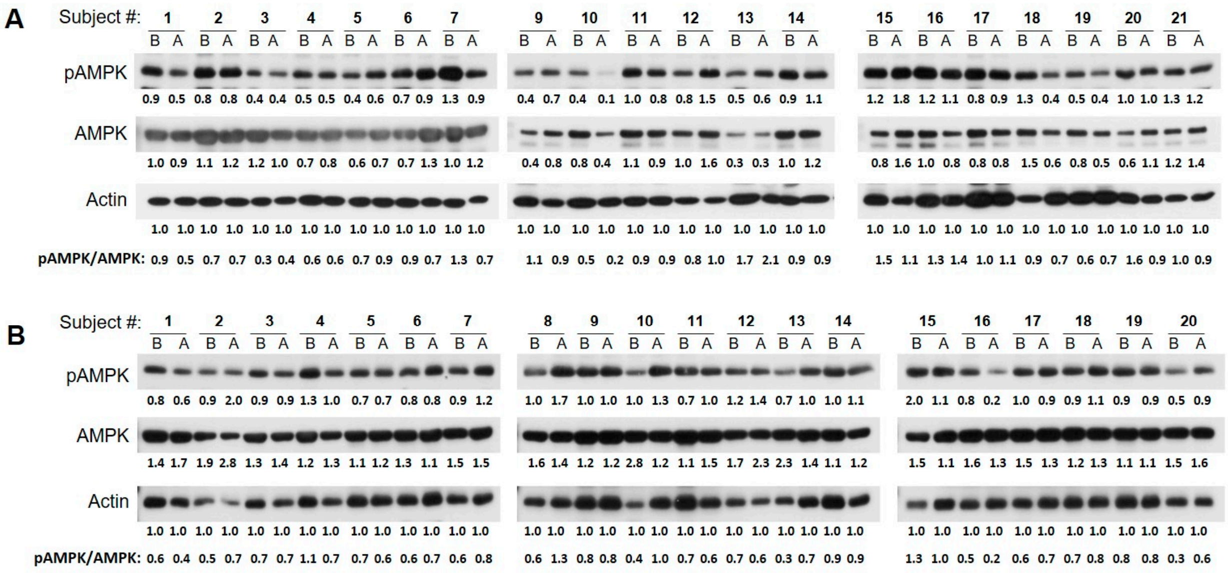

Fig. S2 Lack of effect on nevus AMPK activation in subjects taking ASA. Human subjects were each given ASA daily for one week and nevus samples were obtained both before and after this period. **A**, Western blotting of nevus lysates from subjects before (B) and after (A) one week of daily 325 mg ASA dosing. **B**, Western blotting of nevus lysates from subjects before (B) and after (A) one week of daily 81 mg ASA dosing. Densitometry values normalized to Actin.

Figure S3

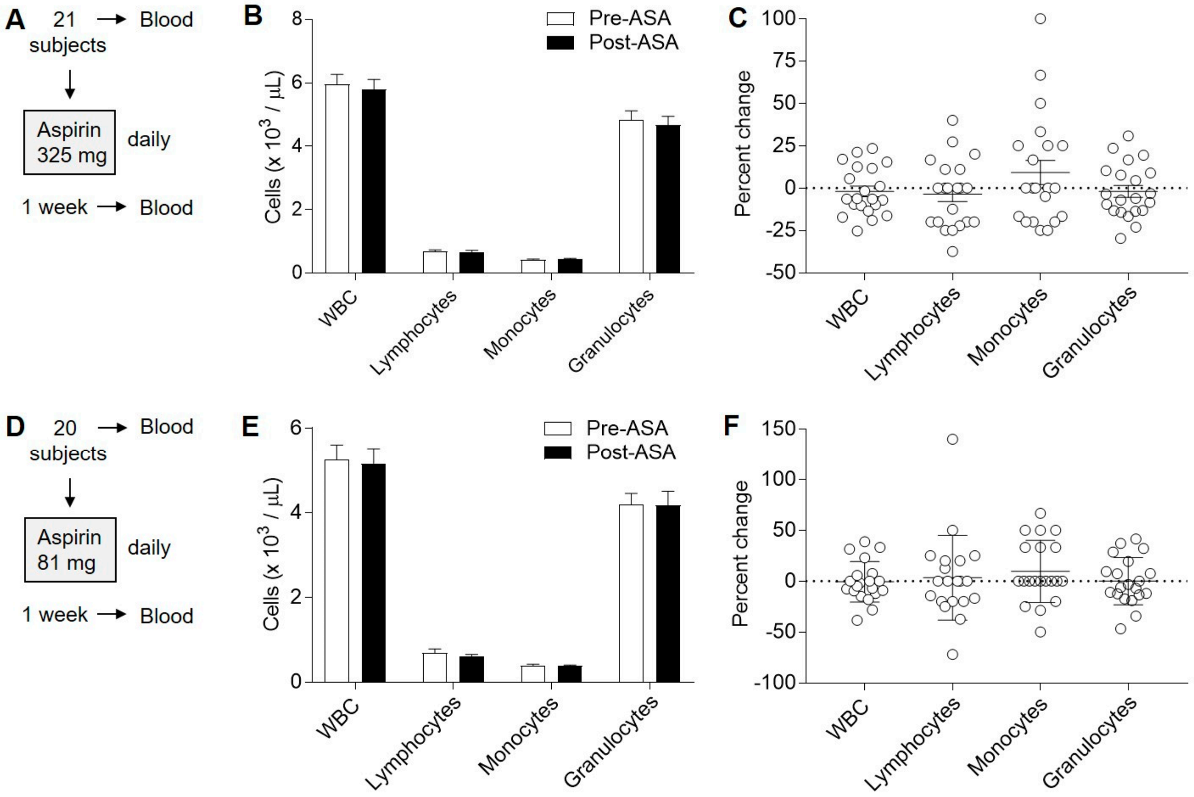

Fig. S3 Lack of effect on blood leukocytes in subjects taking ASA. Human subjects were each given ASA daily for one week and blood samples were obtained both before and after this period. **A**, Protocol for 325 mg dosing. **B**, Levels of white blood cells (WBC) and leukocyte subsets in the 325 mg cohort. Error bars represent SEM. **C**, Percent changes for each leukocyte subset are shown. Error bars represent mean and SD. **D**, Protocol for 81 mg dosing. **E**, Levels of white blood cells (WBC) and leukocyte subsets in the 81 mg cohort. Error bars represent SEM. **F**, Percent changes for each leukocyte subset are shown. Error bars represent mean and SD.

Figure S4

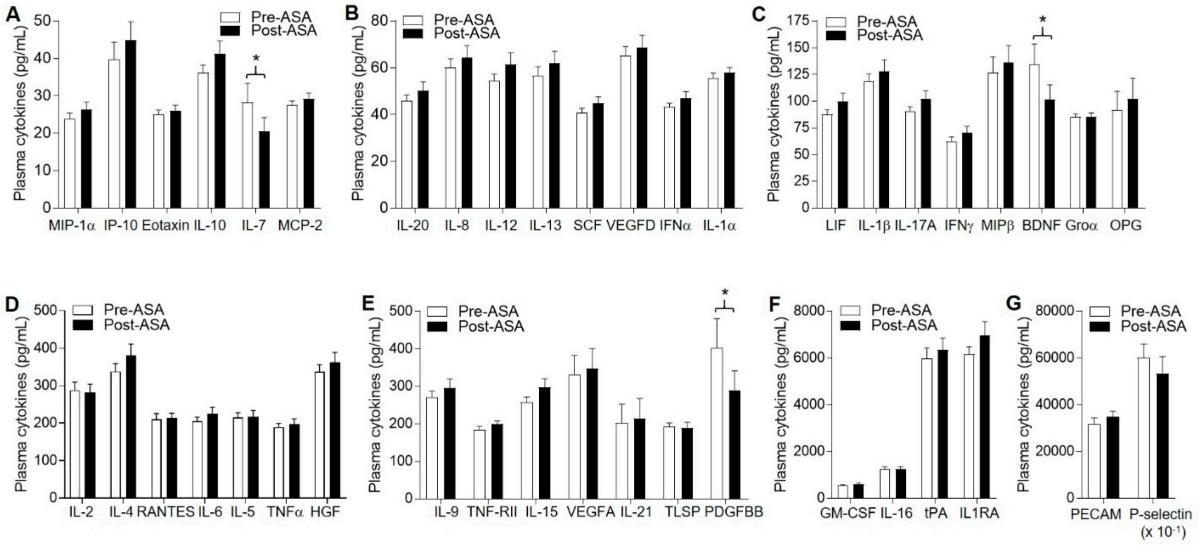

Fig. S4 Effect on plasma cytokine levels in subjects taking 325 mg daily ASA. Human subjects (n=21) were each given 325 mg ASA daily for one week and blood samples were obtained both before and after this period. **A-G**, Levels of indicated cytokines in plasma. Error bars represent SEM. *P<.05, paired t tests.

Figure S5

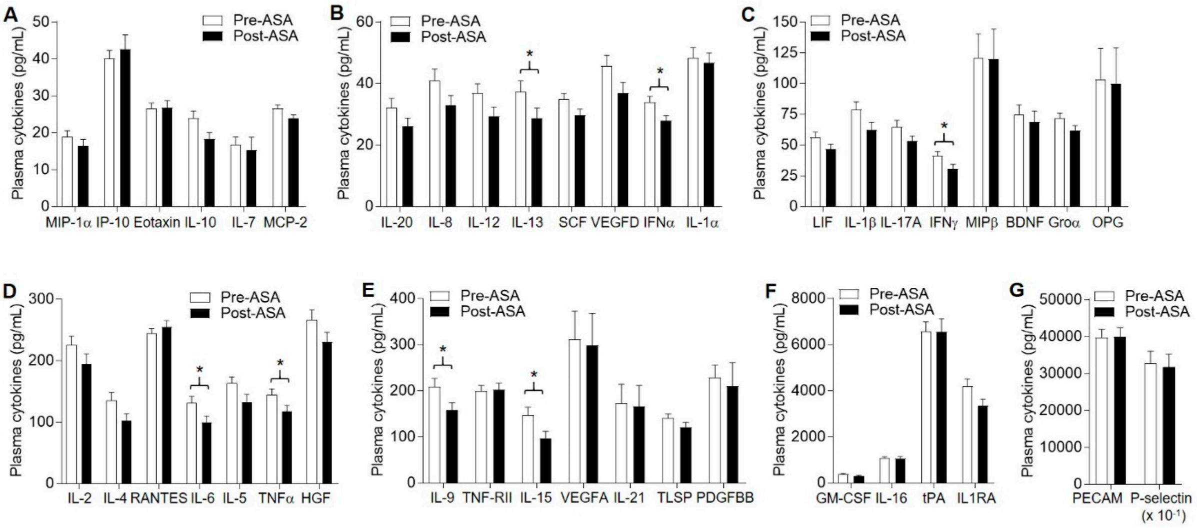

Fig. S5 Effect on plasma cytokine levels in subjects taking 81 mg daily ASA. Human subjects (n=21) were each given 81 mg ASA daily for one week and blood samples were obtained both before and after this period. **A-G**, Levels of indicated cytokines in plasma. Error bars represent SEM. *P<.05, paired t tests.

Figure S6

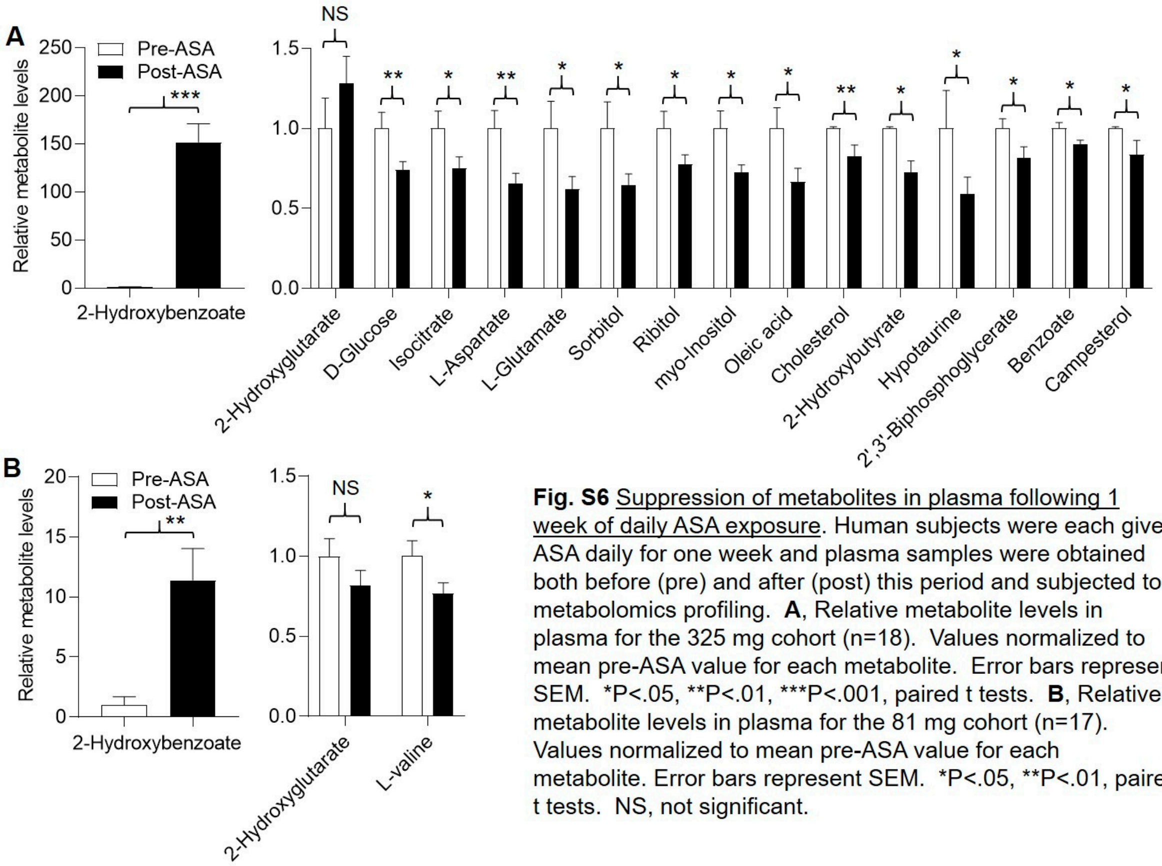

Fig. S6 Suppression of metabolites in plasma following 1 week of daily ASA exposure. Human subjects were each given ASA daily for one week and plasma samples were obtained both before (pre) and after (post) this period and subjected to metabolomics profiling. **A**, Relative metabolite levels in plasma for the 325 mg cohort (n=18). Values normalized to mean pre-ASA value for each metabolite. Error bars represent SEM. *P<.05, **P<.01, ***P<.001, paired t tests. **B**, Relative metabolite levels in plasma for the 81 mg cohort (n=17). Values normalized to mean pre-ASA value for each metabolite. Error bars represent SEM. *P<.05, **P<.01, paired t tests. NS, not significant.
